# Supplementary material for: Colonization of macroalgal deposits by estuarine nematodes through air and potential for rafting inside algal structures
Source: PLoS One. 2021 Apr 15;16(4):e0246723. doi: 10.1371/journal.pone.0246723 (PMC8049275; doi:10.1371/journal.pone.0246723)
Supplement: S1 Table — Replicates were included as a random factor. Pairwise comparisons of the interaction effect are also given. Significant effects are plotted in bold. (DOCX) [file pone.0246723.s001.docx]

**S1 Table. Statistical table of the main effects of the linear mixed model ANOVA with algal structure and inside vs. outside as fixed factors.** Replicates were included as a random factor. Pairwise comparisons of the interaction effect are also given. Significant effects are plotted in bold.

| **factor** | df numerator | df denominator | F | P |
| --- | --- | --- | --- | --- |
| Structure | 1 | 21 | 24.89835 | **0.0001** |
| Inside vs outside | 1 | 21 | 0.74079 | 0.3991 |
| Interaction structure x inside/outside | 1 | 21 | 4.45028 | **0.0471** |
| **Pairwise comparison** | |  | z-value | P |
| receptaculum in vs bladder in | | | 1.855 | 0.204 |
| receptaculum out vs bladder out | | | 4.510 | **< 0.001** |
| receptaculum in vs bladder out | | | 1.630 | 0.308 |
| receptaculum out vs bladder in | | | -1.341 | 0.479 |
